# Laboratory-scale hydraulic fracturing dataset for benchmarking of enhanced geothermal system simulation tools

**DOI:** 10.1038/s41597-020-0564-x

**Published:** 2020-07-08

**Authors:** Paromita Deb, Stephan Düber, Carlo Guarnieri Calo’ Carducci, Christoph Clauser

**Affiliations:** 1 Institute for Applied Geophysics and Geothermal Energy, RWTH Aachen University, Aachen, Germany; 2 Chair of Geotechnical Engineering, RWTH Aachen University, Aachen, Germany; 3 Institute for Automation of Complex Power Systems, RWTH Aachen University, Aachen, Germany

**Keywords:** Geophysics, Research data

## Abstract

Successful design of enhanced geothermal systems (EGSs) requires accurate numerical simulation of hydraulic stimulation processes in the subsurface. To ensure correct prediction, the underlying model assumptions and constitutive relationships of simulators need to be verified against experimental datasets. With the aim of generating laboratory-scale benchmark datasets, a state-of-the-art testing facility was developed, allowing for experiments under controlled conditions. Samples of size 30 cm × 30 cm × 45 cm were subjected to confining stresses while high-pressure fluid was injected into the sample through a pre-drilled borehole, where a saw-cut notch was used to initiate a penny-shaped fracture. Fracture growth and propagation was monitored by measuring pressure data and acoustic emissions detected using 32 seismic sensors. Subsequently, samples were split along the fracture plane to outline the created fracture marked by a red-dyed injection fluid. Finally, a 2D fracture contour was generated using photogrammetry. Presented datasets, accessible via a public repository, include experiments on granite and marble samples. They can be used for verifying and improving numerical codes for field stimulation designs.

## Abstract (summary)

**Table:** 

Measurement(s)	geological fracture • pressure • fluid flow rate • acoustic emission spectrum
Technology Type(s)	visual inspection of rocks after fracturing • pressure transmitters (Keller P33x/1000 bar/80794) • syringe pump • acoustic sensors
Factor Type(s)	rocks (granite and marble)
Sample Characteristic - Environment	laboratory facility

Machine-accessible metadata file describing the reported data: 10.6084/m9.figshare.12490064

## Background & Summary

Engineering a successful stimulation system in the subsurface requires understanding rock response to different pressure conditions. The challenge is to predict as accurately as possible the growth and propagation of fractures in rocks during high-pressure fluid injection, a process called hydraulic stimulation. Several 2D and 3D tools developed by universities and industry are available to numerically simulate growth and predict fracture patterns for any geological condition. Due to the lack of analytical solutions for these coupled, non-linear processes, the accuracy of these simulation tools in solving hydro-mechanical processes can only be assessed by comparing the simulated results against laboratory-scale or field-scale datasets. A code comparison study investigating the capabilities of numerical codes and the quality of the numerical solutions to specific EGS problems is reported in^1^, where real field data was used to benchmark a number of simulators under specific assumptions. Indeed, achieving a well-constrained field-scale hydraulic fracturing dataset is almost impossible due to the unknown geological complexity at depth, which requires major assumptions regarding boundary conditions and subsurface parameters. Therefore, conceivably well-controlled and repeatable laboratory-scale experiments are indispensable for verifying model assumptions and constitutive relationships used by numerical codes to support EGS design.

To obtain meaningful results from laboratory tests, it is necessary to perform experiments on sufficiently large samples so that a stable fracture propagation can be achieved. Experiments of this scale are rather expensive and relatively rare in the geothermal energy sector, especially when performed under true triaxial conditions, i.e. with confining stresses along all the three axes. A non-exhaustive list of true triaxial facilities available worldwide, which can accommodate large samples, is presented in Table 1. However, most of these facilities are often commissioned by oil and gas companies, and therefore their results are not publicly available to the broad scientific community.

**Table 1:** Non-exhaustive list of comparable triaxial set-ups worldwide, list modified from^3^.

Institution	Country	Sample size (*mm*^3^)	Confining Pressure (*MPa*)	Acoustic Emission Data	Background
TerraTek Geomechanics Laboratory (Schlumberger)	USA	762 × 762 × 914 280 × 280 × 381	55 55/21	Yes	Oil
Halliburton	USA	152 × 152 × 254	17	No	Oil
Colorado School of Mines	USA	300 × 300 × 300	12	Yes	Geothermal energy
Earth Science Division, Berkeley	USA	300 × 300 × 450	14/7	Yes	Nuclear storage
Beijing University of Petroleum	China	300 × 300 × 300	28	No	Oil
Jiangsu University of Mining	China	500 × 500 × 500	16	Yes	Coal
Jilin University	China	300 × 300 × 300	20	yes	Geothermal energy, CO2 storage
Tohoku University	Japan	300 × 300 × 300	>12	Yes	Stress measurements
Yamaguchi University	Japan	190 × 190 × 190	6	Yes	Geothermal energy, Oil
Curtin University	Australia	300 × 300 × 300	50	Yes	Oil & Gas
CSIRO Melbourne	Australia	400 × 400 × 400	25	Yes	Oil & Gas
TU Delft	The Netherlands	300 × 300 × 300	39	Yes	Oil
Laboratoire de Mecanique de Lille	France	500 × 500 × 500	70	Yes	Stress measurements

**Table 2:** Specifics of the injection system.

Components	Specifics
Pump	Syringe pump, Teledyne Isco 260 HP
Pressure transmitters	Keller P33x/1000 bar/80794
Inner diameter of the pipes	1.76 mm
Volume of the injection system	12 cm^3^
Initial pump volume	5 cm^3^
Height of the open hole section	30 mm
Radius the borehole	10 mm
Radius of radial notch	17 ± 1 mm
Thickness of radial notch	0.6 mm
Bulk modulus of pump	0.18 GPa
Bulk modulus of injection system (pipes, pressure transmitters and packer)	2.7 GPa

**Table 3:** Data Repository structure.

Contents of Data Repository	Details
AE	scripts for elaborating acoustic emission data
AT	scripts for elaborating active transmission data
DOC	detailed documentation and examples for running the processing code
GEMex02	experimental Data of GEMex02
GEMex03	experimental Data of GEMex03
GEMex04	experimental Data of GEMex04
GEMex06	experimental Data of GEMex06
SystemProperties	experimental boundary conditions and injection system

**Table 4:** Example of the data content of a particular experimental folder (GEMex02).

Name of the file	Format	Detail
GEMex02_InjectionData	.txt	pressure, volume and flow-rate versus absolute time
GEMex02_SensorPositions	.txt	position of acoustic sensors on the surface of the sample
GEMex02_NullTime	.txt	reference time for time conversion from absolute time to time of injection start
GEMex02_RockProperties	.txt	petrophysical and mechanical properties
GEMex02_Fluidproperties	.txt	Injection fluid properties
GEMex02_FractureRadii	.txt	fracture radii obtained from photogrammetry
01-AT_Before_HF	folder	active transmission data acquired before the hydraulic fracturing experiment with sample under confining stresses
02-AE_During_HF	folder	acoustic emission acquired during the hydraulic fracturing experiment with sample under confining stresses
03-AT_After_HF	folder	active transmission data acquired after the hydraulic fracturing experiment with sample under confining stresses
GEMex02-F	.png	image showing the fracture radius of the split sample

Against this background, a large-sample triaxial set-up was developed at RWTH Aachen University under a project funded by BMWi (Federal Ministry of Economics and Technology). The planning and construction of the testing facility, including the analytical and numerical validation of the final experimental procedure, is thoroughly discussed in^2,3^. The set-up allows for experiments on rocks with size 30 cm × 30 cm × 45 cm under defined and controlled boundary conditions, yielding reproducible and repeatable datasets, well-suited for code benchmarking.

In this paper we present hydraulic stimulation datasets from experiments where the borehole axis was parallel to the minimum horizontal stress direction and therefore the plane of crack propagation was parallel to the maximum horizontal stress direction. Investigated rock samples represent the reservoir rock types of a potential EGS site in Mexico. The datasets represent hydraulic stimulation responses in quasi-homogenous and extremely heterogeneous crystalline rock types, such as, a very fine-grained granite and a coarse-grained marble, respectively. Additionally, a dense network of 32 acoustic sensors, comprising of 28 GmuG standard ultrasonic sensors (http://www.gmugmbh.de/prod.html) and 4 Glaser amplitude-calibrated sensors^4,5^, was used to track the fracture propagation in real time.

The validation dataset includes fluid injection rate, pressure in the injection interval, confining stresses, mechanical and petrophysical properties of the rock specimens, properties of the injection fluid, mechanical details of the experimental set-up, and acoustic emission data. Additionally, we provide a Python-based code which can be used for processing the seismic data and visualizing of the experimental results.

## Methods

The main components of the experimental set-up, sample requirements and experimental protocol are described separately in the following sub-sections.

### Sample

Experiments were performed on dense, very low-porosity and low-permeability blocks of granite and marble, cut and polished to final dimensions of 30 cm × 30 cm × 45 cm. A borehole of radius 10 mm was drilled through the horizontal center of the rock, where a circumferential notch with radius 17 ± 1 mm was cut into the borehole wall at a height of z = 22.5 cm to guide the crack initiation.

**Fig. 1:**
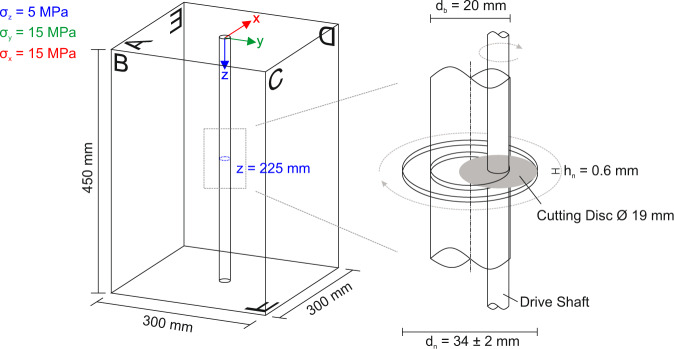
Geometry of the sample, borehole and the notch: naming of sample surfaces (A–F), coordinate system and depth of the notch (z) (left); arrangement for creating a notch in the borehole using cutting disc (right), d_n_ and h_n_ are the diameter and thickness of the notch respectively, d_b_ is the diameter of the borehole..

The tool for cutting the notch, shown in Fig. 1, was developed in-house and described in^2^. It is made of a diamond cutting disc centrally mounted on a long drive shaft. A rotary motor is connected to one end of the shaft, while the other end is mounted in a handle. To cut the notch, the tool is placed centrally in the bore hole of the sample. Then the notch is initially cut in one direction to full depth until the shaft touches the borehole wall. Subsequently, the cutting tool is progressively moved around to create a circular notch on the borehole wall. A guiding aid was used to ensure that the notch depth is 7 ± 1 mm all around.

**Fig. 2:**
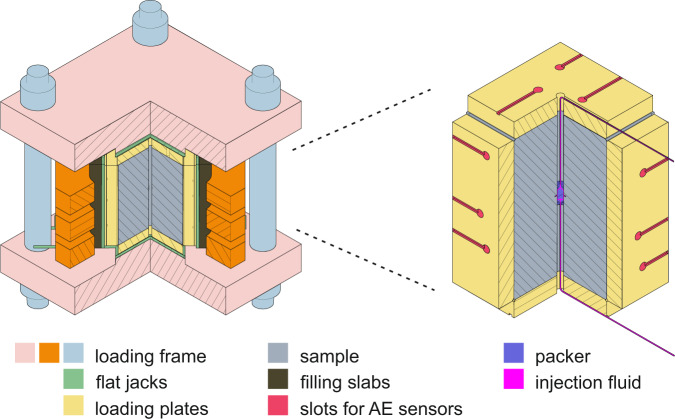
Schematic figure of the triaxial set-up: components of the loading system (left); details of the slots for the acoustic sensors in the loading plates and a cross-section through the sample showing the borehole and the packer configuration (right).

**Fig. 3:**
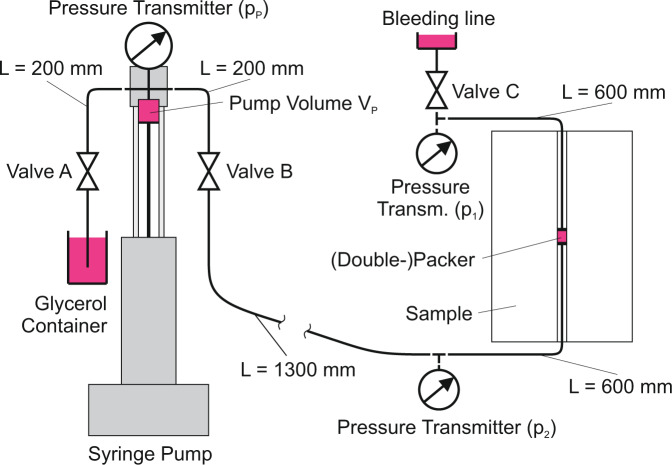
Schematic figure of the injection system: details of valves, sensors and pump position in the hydraulic circuit.

The six surfaces of the samples were alphabetically numbered from A to F. A Cartesian coordinate system was used to systematically describe the samples. The origin of the coordinate system is located in the center of the surface A, the top of the sample. The z-direction corresponds to the borehole axis and points from the upper side A towards the lower side F. The y-axis points from surface E to C and the x-axis is positive from surface B to D (Fig. 1).

### Experimental set-up

The main components of the triaxial set-up for the hydraulic stimulation test are the loading, injection and acoustic data acquisition systems.

#### Loading system

The experiments were performed with samples under three-dimensional confining stress generated by a massive iron loading frame (4600 kg) supported by stiff abutments. Desired stress conditions were achieved by pressurizing metal flat-jacks interleaved between the loading frame and thick iron loading plates leaning against the faces of the sample (Fig. 2). In order to account for the flat-jack rigidity at the edges, their sizes exceed the loading plate dimensions at each side by 10 mm (A, F: 320 × 320 mm; B, C, D and E: 320 × 470 mm), ensuring that the whole area of the sample is uniformly loaded. Teflon films with thickness 1 mm were inserted between each sample face and relative loading plate to reduce the iron-to-rock friction. The edges of the load plates facing the sample were also chamfered to avoid contact between contiguous elements. Circular slots were drilled in the loading plates for accommodating the acoustic sensors, thus ensuring a direct contact with the sample.

#### Injection system

The injection system is composed of a pump, pipes, pressure sensors, a packer and injection fluid. In the center of the 45 cm long borehole, a packer was used to isolate an open-hole section of 30 mm that defines the injection interval (Fig. 3). The packer is terminated by threaded washers that once tightened, press the O-ring against the borehole wall sealing the injection interval from the rest of the borehole and fixing the packer in its position. Measurements were performed in separate experiments, which also allowed for the determination of the bulk modulus (compressibility) of both injection system and pump. Table 2 summarizes the different parameters of the injection system.

The liquid tank valve A and the bleeding line valve C (Fig. 3) were closed during the experiment and the volume of the pump cylinder is reduced to pressurize the injection system. The pressure was measured using two pressure transmitters *p*1 and *p*2 located at the upstream and downstream sections of the injection interval. In order to account for fluid viscosity and improve the measurement accuracy, the pressure of the injection interval was assumed to be the average of *p*1 and *p*2.

The injection fluid used in the experiment is a mixture of 98% glycerol and 2% ink. The red ink was added to the injection fluid to mark the propagation of the fracture and measure the fracture radius after the sample is split along the fracture plane. The dynamic viscosity of the injection fluid is highly temperature-dependent and therefore characterized in a separate test for the temperatures of the experiments using a rotary rheometer. The viscosity values of the injection fluid were then derived for each experiment from the temperature recorded during the experiment. The air content in the injection system was estimated based on the method described in^6^.

#### Acoustic data acquisition system

The acoustic data are acquired using the GMuG data acquisition system (http://www.gmugmbh.de/prod.html), which can operate both in active transmission (AT) and acoustic emission (AE) mode, i.e. in response to a pressure impulse generated actively and seismicity induced due to injection, respectively. The system comprises of piezoelectric sensors, pre-amplifiers, a low-noise multi-channel amplifier and a high-voltage amplifier for pressure impulse generation. The piezoelectric element has a circular contacting point with a diameter of 8 mm, and operates in the bandwidth from 20 kHz to 1 MHz both as transmitter and receiver. Each sensor, with outer body diameter 20 mm, has a defined allotted slot in the loading plates, where a tuneable spring is used to generate a constant and repeatable pressure between the specimen and the sensor. A total of 32 acoustic sensors are distributed over the surface of the sample in non-redundant positions for maximum azimuthal coverage. The original configuration was modified replacing the GMuG-type transducers in the channels (9, 15, 20 and 30) with four Glaser-type conical piezo-electric sensors, laboratory-standard displacement sensors characterized by a quasi-flat wide-band response between 50 kHz and 1 MHz ^4^.

The control unit consists of a dedicated PC equipped with four M2i3122 12-bit 8-channel simultaneous sampling analog-to-digital (ADC) cards and one M2i6011 14-bit digital-to-analog (DAC) card synchronized via M2i Star-Hub technology. The data acquisition was controlled via GMuG software, which allows the configuration of each sensor channel setting and stores the acquired data in binary format both in the standard SEG-Y and in a proprietary GMuG format.

The data collected in AT mode were used for determining average P-wave velocity in the sample. Experiments were performed by stimulating the sample using one sensor at a time as a transmitter, while all the other sensors acted as receivers, generating a total of 868 records: 28 transmitters (excluding Glaser sensors) times 31 acoustic receivers. The transmitter generates a pressure pulse which is obtained by the modulation of a carrier signal at 1 MHz with a cos^4^ envelope function. The P-wave arrival time *t*^*p*^ was then calculated using the Akaike Information Criterion (AIC) ^7^ over the time series *y*(*n*) of length N and sampling period Δ*t* as

$${t}^{p}=\Delta t\cdot arg\mathop{{\rm{\min }}}\limits_{k}AIC\left(k\right),\,{\rm{with}}$$

$$AIC\left(k\right)=k\cdot \log \left(var\left(y\left(1\,:k\right)\right)\right)+\left({\rm{N}}-k-1\right)\cdot \log \left(var\left(y\left(k+1\,:{\rm{N}}\right)\right)\right).$$

Once the first arrivals are picked for every transmitter-receiver pair, their distance was plotted against the P-wave arrival time. A linear regression of all the events provided the average P-wave velocity in the sample which is the value that minimises the sum of squared errors. Signals corresponding to transmitter-receiver pairs which were on the same side of the sample were discarded in the linear regression to avoid false picks related to surface waves.

The data collected in AE mode were used for determining the position of the acoustic source induced during the fracturing of the sample. When a signal on any of the channels exceeds a defined voltage threshold, it triggers the synchronized acquisition in all of the 32 sensors. The signals were sampled at a rate of 10 MHz over a time window of 4096 samples equivalent to 410 µs, with a pre-trigger interval equal to 30% of the window width. For a given acoustic event, the first arrival ${t}_{i}^{p}$ for a sensor *i* was picked using the same AIC criterion as in the case of active transmission experiments. Since the localisation of the seismic events requires the minimisation of a four-dimensional error function *e*(*x*, *y*, *z*, *t*), at least four sensors must detect the event. If this condition is satisfied, then the position of the event (*x*_*E*_, *y*_E_, z_*E*_) and the actual event time *t*_0_ is obtained from the information on the coordinates of the sensors (*x*_i_, *y*_*i*_, *z*_*i*_), the measured first arrival ${t}_{i}^{p}$ and the estimated P-wave propagation speed *v* in the specimen.

$$e(x,y,z,t)=\sum _{{N}_{Ch}}{\left|\left(\frac{\sqrt{{\left({x}_{i}-{x}_{E}\right)}^{2}+{\left({y}_{i}-{y}_{E}\right)}^{2}+{\left({z}_{i}-{z}_{E}\right)}^{2}}}{v}+{t}_{0}\right)-{t}_{i}^{p}\right|}^{2}$$

$$[{x}_{E},{y}_{E},{z}_{E},{t}_{0}]={\rm{\arg }}\mathop{\min }\limits_{(x,y,z,t)}\left(e\right)$$

The ${\rm{\arg }}min(e)$ was obtained using the fminsearch method, which allows the minimisation of a multi-dimensional function through the derivative free Nelder-Mead simplex method^8^.

### Experimental protocol

**Fig. 4:**
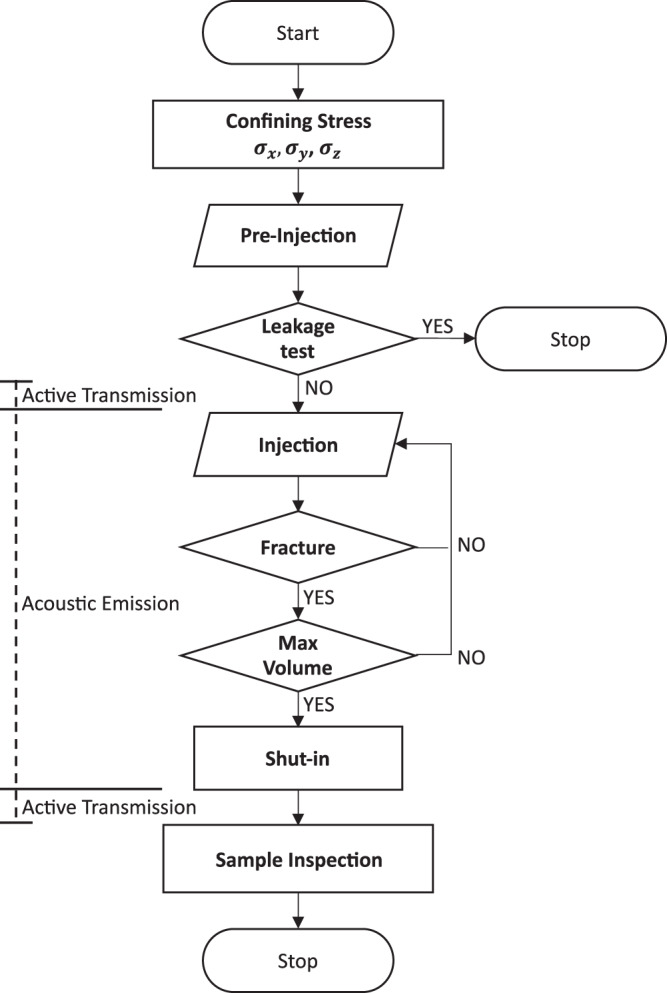
Experimental protocol steps: simplified flow diagram of the protocol explained in the Methods section.

**Fig. 5:**
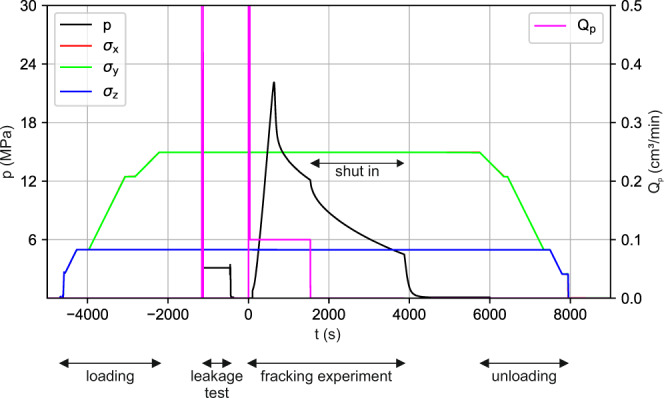
Experiment stages: applied confining stresses σ_x_, σ_y_, and σ_z_ (MPa), pressure p (MPa) and flow rate Q_p_ (cm^3^/min).

Details of the experimental protocol steps are summarised below, whereas the salient stages are graphically depicted in Fig. 4. Example of a pressure response curve versus injection rate during different stages of an experiment is shown in Fig. 5.

**Fig. 6:**
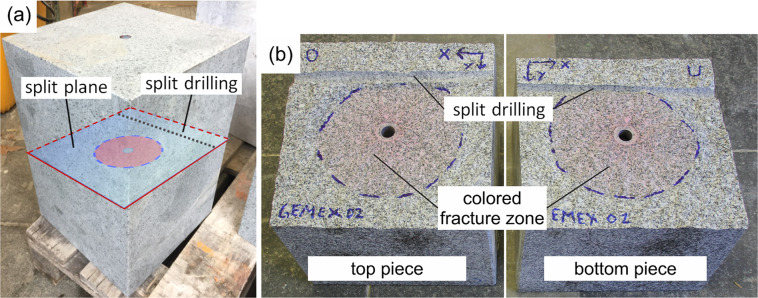
Visual inspection of created fracture: splitting of the sample along fracture plane (**a**), demarcation of the fracture diameter based on the extent of spread of the colored ink (**b**).

- The confining stresses (${\sigma }_{x}={\sigma }_{y}=15\,{\rm{MPa}}$, ${\sigma }_{z}=5\,{\rm{MPa}}$) are applied on the sample.
- All the valves A, B and C are opened and the injection fluid is circulated through the system (from the glycerol container to the bleeding line) to de-air the pipelines and saturate the injection interval (Fig. 3).
- A leakage test is carried out to assess the tightness of the injection system. Valves A and C are closed and the injection system is pressurized to 3 MPa for approximately 600 *s* (Fig. 5). The required fluid volume which has to be injected into the borehole to maintain constant pressure is determined and used as an indicator of the tightness of the system.
- The active transmission experiment is performed to determine the average P-wave velocity of the sample before the fracturing event.
- The fluid volume *V*_0_ stored in the pump is reduced to 5 cm^3^ and the injection of fluid into the sample starts. This time instant is referred to as null time (*t* = 0s) for all recorded data. Simultaneously, the acoustic data acquisition system is configured in acoustic emission mode to begin acquisition of induced seismic events.
- As injection continues, the pressure in the fluid system builds up linearly until the fluid-pressure overcomes the minimum principal stress and the tensile strength of the rock, and the fracture initiates^9^. From this point onwards, the pressure increase until the peak pressure (also called breakdown pressure) is a function of the viscosity of the injection fluid and is monitored using the pressure rate.
- Once the breakdown pressure is reached, only a pre-defined fluid volume increment Δ*V*_*p*_ is further injected into the system to curtail the fracture growth. Afterwards the pump is stopped and the experiment enters the “shut-in” phase.
- During shut-in, the pressure decreases gradually until it falls below the minimum confining stress $\left({\sigma }_{z}=5\,{\rm{MPa}}\right)$, following which the pressure in the injection interval is released.
- The acoustic emission recording is stopped and an active transmission experiment is repeated to evaluate the change in P-wave velocity after fracture formation and propagation.
- The loading frame is disassembled and the sample is removed from the experimental set-up to be split along the fracture plane by means of a second hole drilled perpendicular to the original borehole (Fig. 6(a)).
- The extent of the fracture affected zone is demarcated by the spread of the red dye used in the injection fluid and the fracture radius is measured (Fig. 6(b)).

**Fig. 7:**
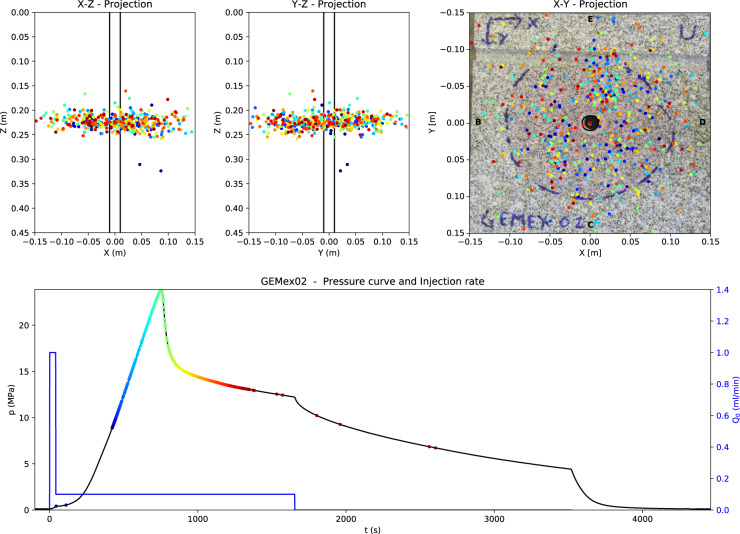
Processed output of a hydraulic fracturing experiment: Final outcome from the processing of GEMex 02 using the provided Python code; localised acoustic emission events in X-Z (top-left) and Y-Z (top-center) projections; photograph of the specimen split along the fracture plane with superimposed localised events and outline (blue) of the fracture (top-right); pressure and injection rate versus time and acoustic emission events with time proportional color scale (bottom).

## Data Records

All experimental data are published via Zenodo Repository^10^. The data repository structure is shown in Table 3. The data structure within each experimental folder is presented in Table 4.

The acoustic emission data are stored in three sub-folders for each experiment. Two folders contain data from active transmission experiments conducted before and after fracturing while the sample is under confining stress, and the third one consists of induced seismicity data recorded during fracturing. Within each of these folders, seismic data are provided in binary format both in GmuG format and SEGY standard format.

## Technical Validation

The engineering details and the experimental system validation are discussed in details in^2,3^. The final adopted experimental procedure is the result of numerous preliminary experiments, which established the validity and repeatability of the measurements^2,3^.

Results obtained from the processing of one of the experiments performed in a granite specimen (GEMex 02), are reported in Fig. 7. The expected stages of the fracturing process can be identified in the pressure profile: pressure rise, breakdown pressure, fracture propagation, and, finally, pressure decay. Pressure increases linearly with the applied constant injection rate to a point called fracture-initiation pressure, where the trend becomes non- linear. The exact time position of this point in the pressure curve cannot be directly identified by visual inspection, but needs to be calculated from the pressure rate curve (*dp*/*dt*). Further increase of pressure, until the breakdown pressure *p*_*b*_, is theoretically a function of the injection fluid viscosity and pressurization rate. In most of our experiments, the fracture initiation pressure is equivalent to the breakdown pressure, after which the pressure decays rapidly. The growth of the fracture is limited by injecting only a limited volume of fluid.

Acquired data, besides being qualitatively consistent with theoretical pressure responses^11^, are also characterized by the accuracy of the measuring instruments, 0.05 MPa for the pressure and 0.5% of the reading for the flow rate, respectively, and a fluid volume step resolution of 16.6 × 10^−6^ ml.

In addition to the pressure profile, the localized acoustic emission events processed using the code provided are also plotted in Fig. 7. The exact localization of events depends strongly on the specific algorithm used for seismic data processing. Although simple in the implementation, this method is very sensitive to errors in the P-wave time of arrival, which are reflected in a significant uncertainty in the AE source localization. More robust techniques should be incorporated to improve the localization accuracy. However, the processed results using the basic code provided within the repository already correlate well with the spread of the dyed injection fluid which delineates the fracture-affected zone (Fig. 7).

## Usage Notes

An example of code execution is provided in the DOC folder (see Table 3) together with flow-diagrams for the most complex scripts as a short documentation. For best compatibility of the Python code, the use of Microsoft Windows environment is recommended.

## Data Availability

Python scripts for basic processing of seismic data and aggregate visualization of both acoustic events and pressure profiles are published in the data repository. Requirements for scripts execution are Python 2.7 and the additional modules *numpy* and *matplotlib*. The code in the repository relies on methods widely available in literature, and is therefore provided “as-is”, with no other warranties, expressed or implied, of correctness and completeness. It is not to be considered as a software for professional data elaboration but rather as an example in support of the research community to develop specific codes by providing a starting template. Users are encouraged to view the code and make necessary changes as required.
